# Kounis syndrome and systemic mastocytosis: one step from elective lipoma resection to cardiac arrest

**DOI:** 10.1186/1710-1492-10-S1-A22

**Published:** 2014-03-03

**Authors:** Marina Lerner, Raveen Pal, Rozita Borici-Mazi

**Affiliations:** 1 Department of Respirology, McMaster University, Hamilton, ON, Canada, L8S 4L8; 2 Department of Cardiology, Queen’s University, Kingston, ON, Canada, K7L 3N6; 3 Department of Allergy and Immunology, Queen’s University, Kingston, ON, Canada, K7L 3N6

## Background

Kounis Syndrome manifests as unstable vasospastic or nonvasospastic angina and is caused by the release of inflammatory mediators following an allergic reaction. A variety of causes have been reported including antibiotics, general anesthetics, latex, insect stings, drug eluting stents, etc. To the best of our knowledge, Kounis syndrome in the setting of systemic mastocytosis has not been previously published. We describe the case of a 52- year- old male, who developed Kounis syndrome perioperatively and was subsequently diagnosed with systemic mastocytosis.

## Methods

Case report and literature review.

## Results

A 52-year-old man was brought to operating room for an elective lipoma resection. Shortly after receiving cefazoline intravenously he developed generalized skin pruritus, flushing and dyspnea. He was treated with intravenous Benadryl and the lipoma site was infiltrated with combination of local anesthetics. He became increasingly agitated, hypoxic and the monitor showed development of ST elevation followed by ventricular fibrillation. The patient sustained a cardiac arrest and required full resuscitation. Return of circulation was achieved and ST segment elevation resolved within minutes of treatment with epinephrine. The procedure was abandoned. He was transferred to our hospital and subsequent angiography showed mild coronary artery disease. A transthoracic ECHO yielded a LVEF of 70% with no wall motion abnormality. His past medical history was remarkable for OSA, hypertension, dyslipidemia, obesity and allergic rhinitis. He reported allergies to insect stings and Terramycin. Blood work obtained one week after the episode showed an elevated baseline tryptase level of 29.3 ng/ml suggesting ongoing mast cell degranulation. He was assessed in Allergy Clinic 3 months later and reported a few years history of symptoms suggestive of Systemic Mastocytosis. His tryptase level was 31.2 ng/ml. RAST for penicillin allergens was negative. Skeletal survey was negative for lytic bone lesions. A working diagnosis of systemic mastocytosis was made and further confirmatory tests are pending. He was started on a combination H1 and H2 antihistamines, leukotriene antagonist and mast cell stabilizer.

## Conclusion

Kounis syndrome is one of the manifestations of anaphylactic reaction. This case report emphasizes the importance of recognizing Kounis syndrome in the setting of anaphylaxis. Effective management of Kounis syndrome should focus on the investigation and treatment of the acute coronary event as well as suppression of the allergic reaction. The flare of underlying systemic mastocytosis due to perioperative stress, rather than a true allergic reaction to cephalosporin, could have caused this reaction; however, further investigations are required.
